# Impaired health-related quality of life in children and adolescents with chronic conditions: a comparative analysis of 10 disease clusters and 33 disease categories/severities utilizing the PedsQL™ 4.0 Generic Core Scales

**DOI:** 10.1186/1477-7525-5-43

**Published:** 2007-07-16

**Authors:** James W Varni, Christine A Limbers, Tasha M Burwinkle

**Affiliations:** 1 Department of Pediatrics, College of Medicine, Department of Landscape Architecture and Urban Planning, College of Architecture, Texas A&M University, 3137 TAMU, College Station, TX 77843-3137, USA; 2 Department of Psychology, College of Liberal Arts, Texas A&M University, College Station, TX 77843-3137, USA; 3 The Children's Hospital at Scott & White, Department of Pediatrics, College of Medicine, Texas A&M University Health Science Center, 2401 South 31st Street, Temple, TX 76508, USA

## Abstract

**Background:**

Advances in biomedical science and technology have resulted in dramatic improvements in the healthcare of pediatric chronic conditions. With enhanced survival, health-related quality of life (HRQOL) issues have become more salient. The objectives of this study were to compare generic HRQOL across ten chronic disease clusters and 33 disease categories/severities from the perspectives of patients and parents. Comparisons were also benchmarked with healthy children data.

**Methods:**

The analyses were based on over 2,500 pediatric patients from 10 physician-diagnosed disease clusters and 33 disease categories/severities and over 9,500 healthy children utilizing the PedsQL™ 4.0 Generic Core Scales. Patients were recruited from general pediatric clinics, subspecialty clinics, and hospitals.

**Results:**

Pediatric patients with diabetes, gastrointestinal conditions, cardiac conditions, asthma, obesity, end stage renal disease, psychiatric disorders, cancer, rheumatologic conditions, and cerebral palsy self-reported progressively more impaired overall HRQOL than healthy children, respectively, with medium to large effect sizes. Patients with cerebral palsy self-reported the most impaired HRQOL, while patients with diabetes self-reported the best HRQOL. Parent proxy-reports generally paralleled patient self-report, with several notable differences.

**Conclusion:**

The results demonstrate differential effects of pediatric chronic conditions on patient HRQOL across diseases clusters, categories, and severities utilizing the PedsQL™ 4.0 Generic Core Scales from the perspectives of pediatric patients and parents. The data contained within this study represents a larger and more diverse population of pediatric patients with chronic conditions than previously reported in the extant literature. The findings contribute important information on the differential effects of pediatric chronic conditions on generic HRQOL from the perspectives of children and parents utilizing the PedsQL™ 4.0 Generic Core Scales. These findings with the PedsQL™ have clinical implications for the healthcare services provided for children with chronic health conditions. Given the degree of reported impairment based on PedsQL™ scores across different pediatric chronic conditions, the need for more efficacious targeted treatments for those pediatric patients with more severely impaired HRQOL is clearly and urgently indicated.

## Background

Advances in biomedical science and technology have resulted in dramatic improvements in the healthcare of pediatric chronic conditions. Many children who either died early in life or were institutionalized are now living well into adulthood and functioning in the community [1]. With this improvement in life status, health-related quality of life (HRQOL) issues have become more salient, particularly given that this survival is accompanied by significant ongoing healthcare needs related to their chronic condition [2]. Simply surviving is not sufficient; the quality of survival has emerged as a fundamental focus of comprehensive healthcare [3].

The last decade has evidenced a dramatic increase in the development and utilization of pediatric HRQOL measures in an effort to improve patient health and well-being and to determine the value of healthcare services [3,4]. A generic HRQOL instrument must be multidimensional, consisting at the minimum of the physical, psychological (including emotional and cognitive), and social health dimensions delineated by the World Health Organization [5,6]. While disease-specific measures may enhance measurement sensitivity for health domains germane to a particular chronic condition, the utilization of a generic HRQOL instrument enables comparisons across chronic conditions and benchmarking with healthy population samples [7-10].

Although a number of studies have investigated the generic HRQOL of adult patients across numerous chronic conditions, typically utilizing the SF-36 [11,12], there has been a relative absence of similar studies which have studied pediatric generic HRQOL across multiple pediatric chronic conditions utilizing the same measurement instrument in which both pediatric patient self-report and parent-proxy reported are obtained [4,13].

A study by Sprangers et al. [14] of generic HRQOL (SF-36) across numerous adult chronic conditions serves as a useful analytic model. These investigators aggregated *disease categories *(e.g., rheumatoid arthritis, osteoarthritis) into 13 *disease clusters *(e.g., musculoskeletal conditions). We modified this overall scheme given the different nature and prevalence of pediatric chronic conditions. Additionally, to identify the specific effects of each condition on HRQOL, we excluded patients with comorbid conditions [15] (except for the psychiatric sample in which this was not possible), and included only patients with physician-diagnosed conditions. We further analyzed the effects of disease severity on HRQOL.

The objectives of the present study were to compare generic HRQOL across ten pediatric chronic disease clusters and 33 disease categories/severities utilizing the PedsQL™ 4.0 Generic Core Scales from the perspectives of pediatric patients and parents. We benchmarked each of the disease clusters to healthy children data.

## Methods

### Selection of samples

Ten disease clusters were composed with physician-diagnosed chronic conditions. These ten disease clusters, which were compiled from a number of separate studies [16-31], consisted of over 2,500 pediatric patients and comprised some of the more prevalent pediatric chronic conditions. For several disease clusters only one disease category was available (e.g., asthma). For those disease categories, we examined disease severity. A sample of over 9,500 healthy children derived from two separate studies [32,33] was included to benchmark the chronic conditions to healthy child data.

### Procedures

Data collection across the chronic condition and healthy samples took place during a 7 year period from 2000 to 2006. For in-person mode of administration, research assistants obtained written parental informed consent and child assent. The PedsQL™ was self-administered for parents and for children ages 8 to 18 and interview-administered for children ages 5 to 7 and in situations in which the child was unable to read or write as a consequence of either physical or cognitive impairment. For telephone administration, parents of children ages 2 to 18 were called by a research assistant who explained the study, and obtained verbal parental informed consent. Child assent was obtained for children ages 5 to 18 years. The research assistant verbally administered the PedsQL™ individually to the parent and their child. If the child was not home at the time of the initial call, the research assistant arranged for a call at another time. For mail mode of administration, parents and children ages 8–18 were instructed to complete the PedsQL™ separately, while parents of children ages 5–7 were instructed to assist their child in completing the survey after completing the proxy-report. These research protocols were approved by the appropriate local Institutional Review Boards (IRBs).

### Samples

Table 1 presents sociodemographic characteristics for each sample.

**Table 1:** Sociodemographic Characteristics.

	Healthy	Diabetes	Cardiac	Asthma^a^	Asthma^b^	ESRD	Psych.	Cancer	Rheum.	CP	Obesity^c^	GI
Characteristics												

Self-Report:												
Mean Age^¥ ^(SD)	9.79 (3.15)	13.65 (3.33)	11.68 (3.62)	9.58 (1.65)	13.62 (1.28)	13.54 (3.77)	N/A	11.49 (4.25)	12.56 (3.45)	10.42 (3.92)	12.10 (3.00)	11.57 (3.27)
Age Range^¥^	5.00–18.10	5.00–18.99	5.00–18.85	7.00–12.00	12.00–18.00	5.00–18.99		5.00–18.99	5.01–18.90	5.00–18.00	5.00–18.34	5.00–18.00
Self-Report:												
Gender												
Male	2836 (51.5%)	134 (44.7%)	173 (60.3%)	93 (57.1%)	69 (54.8%)	47 (55.3%)	N/A	220 (55.8%)	63 (18.8%)	40 (50.6%)	57 (54.0%)	132 (47.1%)
Female	2671 (48.5%)	166 (55.3%)	114 (39.7%)	68 (41.7%)	52 (41.3%)	21 (24.7%)		173 (43.9%)	255 (75.9%)	37 (46.8%)	49 (46.0%)	148 (52.9%)
Total Sample:												
Mean Age ^¥ ^(SD)	7.83 (3.99)	13.20 (3.83)	9.21 (4.73)	9.56 (1.65)	9.04 (3.62)	12.62 (4.54)	11.30 (3.20)	9.64 (4.93)	11.44 (4.28)	8.08 (4.33)	12.10 (3.00)	11.43 (3.37)
Age Range^¥^	2.00–18.10	3.00–18.99	2.07–18.85	7.00–12.00	2.00–18.00	2.46–18.99		2.01–18.99	2.38–18.90	2.00–18.00	3.04–18.34	2.00–18.00
Total Sample:												
Gender												
Male	4918 (51.4%)	149 (45.0%)	246 (57.7%)	93 (56.4%)	251 (54.2%)	52 (54.2%)	185 (59.7%)	326 (57.0%)	80 (20.4%)	134 (54.7%)	57 (54.0%)	136 (47.4%)
Female	4647 (48.6%)	180 (54.4%)	180 (42.2%)	69 (41.8%)	181 (39.1%)	25 (26.0%)	125 (40.3%)	245 (42.8%)	293 (74.6%)	107 (43.7%)	49 (46.0%)	151 (52.6%)
Total Sample:												
Race/Ethnicity												
White	14.1%	46.5%	78.4%	34.5%	26.8%	38.5%	N/A	22.0%	44.8%	44.9%	26.4%	71.1%
Hispanic	61.7%	25.1 %	7.7%	5.5 %	24.2%	18.8%	N/A	41.1%	19.1 %	33.9 %	59.4%	15.7%
Black	2.5%	10.6 %	8.9%	8.5%	28.7%	16.7%	N/A	3.0 %	3.8 %	4.9 %	7.5%	10.1%
Asian/Pacific Islander	10.9%	8.8 %	2.1%	0.6 %	N/A	1.0 %	N/A	3.8 %	4.1 %	5.3 %	0.9%	0.7%
American	0.4%	1.5 %	0.0%	0.0%	N/A	0.0%	N/A	0.0%	0.0%	0.8 %	0.0%	0.4%
Indian/Alaskan Other	0.5%	6.0%	0.7%	3.0%	20.3%	3.1%	N/A	5.8 %	2.0 %	4.1 %	5.7%	2.4%
Total Sample:												
Mean SES												
Index^‡ ^(SD)	N/A⟡	42.72* (14.30)	42.13* (13.45)	42.11*^~ ^(12.04)	N/A	36.13^§ ^(13.57)	N/A^†^	35.43^§¤ ^(17.82)	44.73* (14.20)	42.48* (14.26)	35.60^§ ^(14.50)	N/A

Note: ^¥ ^= Mean age and range in years.

^‡ ^= Total Sample Mean SES Index based on the Hollingshead SES Index.

N/A = Not Available.

^⟡ ^= Mean SES was unavailable for this sample, although the statewide SCHIP sample was representative of low income families (< 250% of the federal poverty level).

* = Based on the Hollingshead Index, indicates on average a middle-class family SES.

^§ ^= Based on the Hollingshead Index, indicates on average a lower middle-class family SES.

^~ ^= Mean socioeconomic status (SES) was unavailable for families assessed at the American Lung Association summer camp, thus SES value is based on families assessed at the University of Kansas Medical Center (n = 86)

^¤ ^= Mean socioeconomic status (SES) was unavailable for families assessed at Lucile Packard Children's Hospital, Stanford, thus SES value is based on families assessed at Children's Hospital and Health Center, San Diego, and Childrens Hospital Los Angeles (n = 438).

^† ^= SES reported not using Hollingshead SES Index; 33.2 % of the pediatric patients came from low SES families, 30.0 % from medium SES families, and 36.8 % from high SES families.

^a ^= Asthma sample utilized for HRQOL comparisons across disease clusters.

^b ^= Asthma sample utilized for HRQOL comparisons across disease categories.

^c ^= Sociodemographic information for the obesity sample represents the clinical sample of severely obese children (n = 106) used for comparisons across disease categories, given the unavailability of sociodemographic information for the obese and overweight community samples. ESRD equals End Stage Renal Disease. Psych. equals Psychiatric Disorders. Rheum equals Rheumatology. CP equals Cerebral Palsy. GI equals Gastrointestinal Conditions.

### Pediatric asthma sample

The asthma sample (n = 165) utilized for HRQOL comparisons across the disease clusters was derived from the PedsQL™ 3.0 Asthma Module field test [16]. Children and their parents were assessed in-person by a research assistant at the University of Kansas Medical Center and at a summer camp sponsored by the American Lung Association [16]. Since information on patients' asthma severity was unavailable for this sample, a separate asthma sample was utilized for HRQOL disease severity comparisons. This sample (n = 463) was derived from the initial field test of the PedsQL™ 4.0 SF-15, a short version of the PedsQL™ 4.0, and the PedsQL™ 3.0 SF-22 Asthma Module, a short version of the Asthma Module [17]. The PedsQL™ was administered by telephone to 125 adolescents (ages 12–18) and 338 parents of patients with asthma (ages 2–11) who had been seen for asthma care at 1 of 13 geographically dispersed clinics in the United States [17]. Asthma severity included patients with mild intermittent asthma (n = 281, 60.7 %), mild persistent asthma (n = 96, 20.7 %), and moderate to severe persistent asthma (n = 86, 18.6 %).

### Pediatric cancer sample

The cancer sample (n = 572) was derived from the PedsQL™ 3.0 Cancer Module field test (n = 339, 59.3 %) [18], the PedsQL™ Brain Tumor Module field test (n = 99, 17.3 %) [19], and a study of patients with brain tumors (n = 134, 23.4 %) [20]. The sample included patients with brain tumors (n = 257, 44.9 %), acute lymphocytic leukemia (n = 171, 29.9 %), non-Hodgkin's lymphoma (n = 20, 3.5 %), Wilm's tumor (n = 19, 3.3 %), neuroblastoma (n = 16, 2.8 %), Hodgkin's lymphoma (n = 11, 1.9 %), and other cancers (n = 78, 13.6 %). Children and their parents were assessed in-person by a research assistant at hematology/oncology centers at Children's Hospital and Health Center, San Diego, Childrens Hospital Los Angeles, and Lucile Packard Children's Hospital at Stanford [16-18].

### Pediatric cardiac sample

The cardiac sample (n = 426) was derived from the PedsQL™ 3.0 Cardiac Module field test [21] and the PedsQL™ 4.0 field test [22]. Heart disease was categorized as: 1 = mild disease requiring no therapy or effectively treated non-operatively (catheter therapy); 2 = moderate disease surgically corrected (curative) or requiring no therapy; 3 = surgical correction (one or more procedures) with significant residua or need for further surgery; 4 = complex or severe disease, uncorrectable or palliated (includes single ventricle). There were 82 patients in category 1, 143 in category 2, 93 in category 3, and 108 in category 4. Children and their parents were assessed in-person by a research assistant at pediatric cardiology clinics at Cincinnati Children's Hospital Medical Center and Children's Hospital and Health Center, San Diego [21].

### Pediatric cerebral palsy sample

The cerebral palsy sample (n = 245) was derived from the PedsQL™ 3.0 Cerebral Palsy Module field test [23]. The sample included patients with paraplegia (n = 73, 32.0 %), diplegia (n = 69, 30.3 %), and hemiplegia (n = 50, 21.9 %). Children and their parents were assessed in-person by a research assistant at the Cerebral Palsy Clinic at Children's Hospital and Health Center, San Diego, as well as state medical therapy clinics in San Diego County [23].

### Pediatric diabetes sample

The diabetes sample (n = 331) was derived from the PedsQL™ 3.0 Diabetes Module field test [24]. The sample included patients with Type 1 diabetes (n = 236, 71.5 %) and Type 2 diabetes (n = 91, 27.6 %). Children and their parents were assessed in-person by a research assistant at pediatric endocrinology clinics at Childrens Hospital Los Angeles and Children's Hospital and Health Center, San Diego, or via telephone [24].

### Pediatric end stage renal disease sample

The end stage renal disease sample (n = 96) included patients with a renal transplant (n = 45, 46.9 %), and patients receiving hemodialysis (n = 32, 33.3 %) and peritoneal dialysis (n = 19, 19.8 %) [25]. Children and their parents were assessed in-person by a research assistant at Baylor College of Medicine/Texas Children's Hospital, Houston, Texas or Children's Mercy Hospital and Clinics, Kansas City, Missouri [25].

### Pediatric gastrointestinal conditions sample

The gastrointestinal conditions sample (n = 287) included patients who met Rome II criteria for irritable bowel syndrome (n = 123, 42.9%) and functional abdominal pain (n = 82, 28.6%), and patients with a clinical diagnosis of an organic gastrointestinal disorder (n = 82, 28.6%) [26]. Children and their parents were assessed in-person by a research assistant at Scott & White Regional Pediatric Gastroenterology Clinic, Temple, TX, Children's Hospital and Health Center, San Diego, and the Center for Pediatric Irritable Bowel and Motility Disorders, Morristown, NJ [26].

### Pediatric obesity sample

The obesity sample (n = 63) utilized for HRQOL comparisons across the disease clusters was derived from a community sample classified as obese according to Body Mass Index (BMI) cut points [27]. In addition, a clinic sample diagnosed as severely obese (n = 106) [28] and a community sample of overweight children (n = 294) [27] were compared. Children and their parents were assessed in-person by a research assistant at an obesity clinic at Children's Hospital and Health Center, San Diego and in-person in the schools in the state of Victoria, Australia [27].

### Pediatric psychiatric disorders sample

Children diagnosed with a psychiatric disorder by a clinician at a general or university outpatient child psychiatric clinic in The Netherlands, and their parents, were assessed via mailing and in-person at a home visit [29]. The psychiatric disorders sample (n = 310) included patients with attention-deficit and disruptive behavior disorders (n = 107, 48.4 %), anxiety disorders (n = 57, 25.8 %), mood disorders (n = 29, 13.1 %), and pervasive developmental disorders (n = 28, 12.7 %) [29]. Multiple diagnoses were permitted. However, the diagnosis of greatest immediate clinical significance was designated as the child's primary diagnosis by the clinician for the purposes of the study [29].

### Pediatric rheumatology sample

The rheumatology sample (n = 393) was derived from the PedsQL™ 3.0 Rheumatology Module field test [30] and the PedsQL™ Multidimensional Fatigue Scale field test in rheumatology [31]. The sample included patients with juvenile rheumatoid arthritis (n = 105, 26.7 %), fibromyalgia (n = 59, 15.0 %), spondyloarthritis (n = 39, 9.9 %), systemic lupus erythematosus (n = 29, 7.4 %), and other rheumatologic conditions (n = 157, 40.0 %). Children and their parents were assessed in-person by a research assistant at the pediatric rheumatology clinic at Children's Hospital and Health Center, San Diego [31].

### Healthy children sample

The healthy children sample (n = 9566) was derived from the PedsQL™ 4.0 initial field test (n = 730, 7.6 %) [32] and a State Children's Health Insurance Program evaluation (n = 8836, 92.4 %) [33]. Healthy children are those children who were assessed either in physicians' offices during well-child checks and/or whose parents did not report the presence of a chronic health condition. Children and their parents from the PedsQL™ 4.0 initial field test were assessed in-person by a research assistant at Children's Hospital and Health Center, San Diego, or via telephone [32]. Children and their parents from the State Children's Health Insurance Program evaluation were assessed via mail surveys [33].

### Measures

#### The PedsQL™ 4.0 (Pediatric Quality of Life Inventory™ Version 4.0)

The 23-item PedsQL™ 4.0 Generic Core Scales encompass: 1) Physical Functioning (8 items), 2) Emotional Functioning (5 items), 3) Social Functioning (5 items), and 4) School Functioning (5 items), and were developed through focus groups, cognitive interviews, pre-testing, and field testing measurement development protocols [8,32]. The instrument takes approximately 5 minutes to complete [32].

The PedsQL™ Scales are comprised of parallel child self-report and parent proxy-report formats. Child self-report includes ages 5–7, 8–12, and 13–18 years. Parent proxy-report includes ages 2–4 (toddler), 5–7 (young child), 8–12 (child), and 13–18 (adolescent), and assesses parent's perceptions of their child's HRQOL. The items for each of the forms are essentially identical, differing in developmentally appropriate language, or first or third person tense. The instructions ask how much of a problem each item has been during the past one month. A 5-point Likert response scale is utilized across child self-report for ages 8–18 and parent proxy-report (0 = never a problem; 1 = almost never a problem; 2 = sometimes a problem; 3 = often a problem; 4 = almost always a problem). To further increase the ease of use for the young child self-report (ages 5–7), the response scale is reworded and simplified to a 3-point scale (0 = not at all a problem; 2 = sometimes a problem; 4 = a lot of a problem), with each response choice anchored to a happy to sad faces scale [34,35].

Items are reverse-scored and linearly transformed to a 0–100 scale (0 = 100, 1 = 75, 2 = 50, 3 = 25, 4 = 0), so that higher scores indicate better HRQOL. Scale Scores are computed as the sum of the items divided by the number of items answered (this accounts for missing data). If more than 50% of the items in the scale are missing, the Scale Score is not computed. This accounts for the differences in sample sizes for scales reported in the Tables. Although there are other strategies for imputing missing values, this computation is consistent with the previous PedsQL™ peer-reviewed publications, as well as other well-established HRQOL measures [32,36,37]. The Physical Health Summary Score (8 items) is the same as the Physical Functioning Scale. To create the Psychosocial Health Summary Score (15 items), the mean is computed as the sum of the items divided by the number of items answered in the Emotional, Social, and School Functioning Scales.

#### PedsQL™ Family Information Form

The PedsQL™ Family Information Form [32], or survey items adapted from the PedsQL™ Family Information Form, were completed by most parents. The PedsQL™ Family Information Form contains demographic information including the child's date of birth, gender, race/ethnicity, and parental education and occupation information required to calculate the Hollingshead SES index [38].

### Statistical analyses

#### Influence of sociodemographic characteristics

The influence of sociodemographic characteristics (patients' age, gender, race/ethnicity) on the PedsQL™ Total Scale Score were examined for each disease cluster (with the exception of the psychiatric disorders cluster, in which these data were unavailable) using independent samples *t*-tests and analysis of variance with Tukey post-hoc tests.

#### Comparisons across disease clusters and a healthy sample

Mean PedsQL™ scale and summary scores were calculated for each disease cluster and a healthy sample and compared using analysis of variance with Tukey post-hoc tests. Effect size as utilized in these analyses was calculated by taking the difference between each disease cluster mean and the healthy sample mean, divided by the healthy sample standard deviation. Effect sizes for differences in means are designated as small (.20), medium (.50), and large (.80) in magnitude [39].

#### Comparison of disease categories/severities within their respective disease clusters

Mean PedsQL™ scale and summary scores were calculated for each disease category/severity and compared within their respective disease cluster using independent samples *t-*tests when 2 disease categories were being contrasted and analysis of variance with Tukey post-hoc tests when more than 2 disease categories were being contrasted.

## Results

### Influence of sociodemographic characteristics

#### Age

Adolescents in the cardiac cluster reported significantly higher HRQOL than young children, and young children in the asthma and gastrointestinal clusters reported significantly higher HRQOL than older children. Young children and older children in the gastrointestinal cluster reported significantly higher HRQOL than adolescents. Parents reported significantly higher HRQOL for toddlers than older children and adolescents in the diabetes, cardiac, cancer, and rheumatology clusters.

#### Gender

No gender effects were found.

#### Race/ethnicity

No race/ethnicity effects were found for patient self-report. Parents proxy-reported several race/ethnicity differences across 3 disease clusters (diabetes, cardiac, asthma), which did not achieve statistical significance after a Bonferroni correction.

### Comparisons across disease clusters and with a healthy sample

#### Overall HRQOL

Patients and parents across the disease clusters reported significantly lower overall HRQOL in comparison to healthy children and their parents (Tables 2 and 3). The majority of differences for patient self-report demonstrate medium to large effect sizes, while the majority of differences for parent proxy-report demonstrate large effect sizes. Patients in the diabetes cluster self-reported the highest overall HRQOL and parents proxy-reported the highest overall patient HRQOL for the cardiac cluster. Patients and parents in the cerebral palsy cluster reported the lowest overall HRQOL.

**Table 2:** Child Self-Report: Means and Standard Deviations for the PedsQL™ 4.0 Generic Core Scales by Disease Cluster and Comparisons with Healthy Children Scores.

	Healthy^a^	Diab.^b^	GI^c^	Card.^d^	Asthma^e^	Obesity^f^	ESRD^g^	Psych.^h^	Cancer^i^	Rheum.^j^	CP^k^	
Scale	Mean (SD)	Mean (SD) *ES*	Mean (SD) *ES*	Mean (SD) *ES*	Mean (SD) *ES*	Mean (SD) *ES*	Mean (SD) *ES*	Mean (SD) *ES*	Mean (SD) *ES*	Mean (SD) *ES*	Mean (SD) *ES*	Differences

**Self-Report**	(n = 5480)	(n = 300)	(n = 280)	(n = 250)	(n = 162)	(n = 63)	(n = 85)	(n = 296)	(n = 393)	(n = 336)	(n = 77)	

Total Score	83.84 (12.65)	80.35 (12.89) *0.28*	77.79 (13.24) *0.48*	77.47 (14.51) *0.50*	74.85 (16.52) *0.71*	74.00 (14.20) *0.78*	73.97 (15.22) *0.78*	72.20 (12.70) *0.92*	71.97 (16.12) *0.94*	70.35 (17.83) *1.07*	66.85 (16.73) *1.34*	a > b, d, e, g, h, i, j, k, f, c***; b, d > h***; h > k*; b > e, g**; b > i, j, k***; d, c > i, j, k***; e > j*; e > k***; g > k*; i > k*; c > h***;b > f*
Physical Health	87.53 (13.50)	85.89 (13.33) *0.12*	80.80 (13.84) *0.50*	82.28 (15.68) *0.39*	76.51 (18.01) *0.82*	77.50 (17.90) *0.74*	74.73 (20.43) *0.95*	81.20 (14.20) *0.47*	71.97 (21.37) *1.15*	65.99 (23.81) *1.60*	64.40 (22.08) *1.71*	a > d, e, g, h, i, j, k, f, c***; b > h, f, c**; h > e, g*; h, c > i, j, k***; b > e, g, i, j, k ***; d > e, g**; d > i, j, k***; e, f > j, k***; e > i*; g > j, k***; i > j***; i > k**; c > g*
Psychosocial Health	81.87 (14.09)	77.34 (14.62) *0.32*	76.18 (14.63) *0.40*	74.88 (16.10) *0.50*	73.95 (18.35) *0.56*	72.10 (14.10) *0.69*	73.54 (14.80) *0.59*	67.40 (14.70) *1.03*	72.10 (16.31) *0.69*	72.67 (17.07) *0.65*	68.11 (16.52) *0.98*	a > b, k, e, g, h, i, j, k, f, c***; b, d, e, i, j, c > h***; g > h*; b > j**; b > i, k***; d > k**; c > i*; c > k**
Emotional Functioning	79.33 (18.15)	72.37 (19.57) *0.38*	73.95 (18.76) *0.30*	73.78 (20.38) *0.31*	72.93 (22.58) *0.35*	68.60 (18.50) *0.59*	75.16 (18.88) *0.23*	61.30 (19.50) *0.99*	72.20 (20.84) *0.39*	68.32 (22.85) *0.61*	68.60 (22.93) *0.59*	a > b, k, e, h, i, j, k, f, c***; b, d, e, g, i, j, c > h***; d > j*; c > j**
Social Functioning	85.15 (16.76)	85.63 (16.24) *0.03*	84.33 (15.77) *0.05*	78.74 (19.52) *0.38*	78.94 (19.79) *0.37*	72.60 (18.20) *0.75*	78.46 (17.79) *0.40*	73.00 (20.40) *0.72*	75.54 (21.09) *0.57*	80.10 (19.10) *0.30*	70.52 (19.26) *0.87*	a > d, e, h, i, j, k, f***; a > g*; b, j, c > h***; d > h**; e > h*; b > d, i, k***; b > e, j**; b > g*; d > k**; e, j > k***; j > i**; e > k*; c > d**; c > e*; c > i, k, f***; b > f***; j > f*
School Functioning	81.12 (16.45)	74.20 (18.08) *0.42*	70.29 (18.56) *0.66*	72.09 (19.01) *0.55*	70.00 (21.44) *0.68*	75.00 (14.50) *0.37*	66.93 (19.15) *0.86*	67.90 (16.70) *0.80*	68.30 (19.53) *0.78*	69.86 (19.97) *0.68*	65.61 (22.18) *0.94*	a > b, d, e, g, h, i, j, k, c***; b > h***; b > g, j, k**; b > i***; f > k*

Note: *p < .05, **p < .01, ***p < .001 based on analysis of variance with Tukey post-hoc tests.

With a Bonferroni correction for the number of comparisons, p < .005 values should be considered statistically significant.

Higher values equal better health-related quality of life. Disease clusters are ordered from left to right by decreasing overall HRQOL.

ES equals effect size.

Effect size represents the magnitude in the difference between the disease cluster and the healthy children sample.

Effect sizes are designated as small (.20), medium (.50), and large (.80).

Diab. equals Diabetes. Card. equals Cardiac. ESRD equals End Stage Renal Disease. Psych. equals Psychiatric Disorders. Rheum equals Rheumatology. CP equals Cerebral Palsy. GI equals Gastrointestinal Conditions.

**Table 3:** Proxy-Report: Means and Standard Deviations for the PedsQL™ 4.0 Generic Core Scales by Disease Cluster and Comparisons with Healthy Children Scores.

	Healthy^a^	Card.^b^	Diab.^c^	Obesity^d^	GI^e^	ESRD^f^	Asthma^g^	Rheum.^h^	Cancer^i^	Psych.^j^	CP^k^	
Scale	Mean (SD)	Mean (SD) *ES*	Mean (SD) *ES*	Mean (SD) *ES*	Mean (SD) *ES*	Mean (SD) *ES*	Mean (SD) *ES*	Mean (SD) *ES*	Mean (SD) *ES*	Mean (SD) *ES*	Mean (SD) *ES*	Differences

**Proxy-Report**	(n = 9430)	(n = 344)	(n = 307)	(n = 63)	(n = 286)	(n = 95)	(n = 157)	(n = 357)	(n = 561)	(n = 307)	(n = 224)	

Total Score	82.70 (15.40)	79.44 (16.50) *0.21*	76.62 (14.08) *0.39*	75.00 (14.50) *0.50*	72.74 (14.75) *0.65*	69.62 (18.10) *0.82*	68.79 (15.94) *0.90*	68.73 (19.32) *0.91*	68.47 (19.22) *0.92*	66.90 (14.00) *1.03*	51.28 (18.00) *2.04*	a > c, g, f, j, i, h, k, d, e***; a > b**; c, b, e > j***; c > g, i, h, k***; c > f**; b > g, f, i, h, k***; g, f, j, i, h, d, e > k***; b > e***; e > h*; d > j**
Physical Health	84.48 (19.51)	83.11 (18.73) *0.07*	82.02 (17.20) *0.13*	76.30 (17.60) *0.42*	76.00 (16.96) *0.43*	71.21 (24.24) *0.68*	72.68 (18.39) *0.60*	64.05 (25.01) *1.05*	67.55 (25.07) *0.87*	80.00 (17.70) *0.23*	43.19 (27.59) *2.12*	a > j, d**; a > g, f, i, h, k, e***; j > g, f**; j, e > i, h, k***; b, c > g, f, i, h, k***; g > h, k***; f > h*; f, i, h > k***; c > e*; b > e***; d > h, k***; d > i*
Psychosocial Health	81.65 (15.22)	77.36 (17.27) *0.28*	73.67 (15.34) *0.52*	73.90 (15.30) *0.51*	71.00 (15.58) *0.70*	68.85 (17.62) *0.84*	66.83 (16.68) *0.97*	71.27 (18.49) *0.68*	69.12 (18.41) *0.82*	59.90 (15.30) *1.43*	55.91 (16.98) *1.69*	a > b, c, g, f, j, i, h, k, e***; a > d**; b, c, g, f, i, h > j***; c > i**; c > g, k***; b > g, f, i, h, k***; g, f, i, h > k***; e > j, k***; b > e***; d > j, k***
Emotional Functioning	81.31 (16.50)	74.69 (20.45) *0.40*	69.16 (18.54) *0.74*	72.60 (17.80) *0.53*	65.19 (19.28) *0.98*	68.74 (19.69) *0.76*	64.81 (20.21) *1.00*	66.52 (22.27) *0.90*	67.57 (20.70) *0.83*	54.40 (18.70) *1.63*	62.73 (19.55) *1.13*	a > b, c, g, f, j, i, h, k, d, e***; b, c, g, f, i, h, k, d, e > j***; b > c**; c > k**; b > g***; b > i, h, k***; i > k*; b > e***; d > k**
Social Functioning	83.70 (19.43)	82.52 (20.11) *0.06*	81.08 (19.33) *0.13*	73.50 (17.30) *0.52*	80.30 (18.08) *0.17*	73.47 (21.00) *0.53*	73.41 (18.66) *0.53*	76.47 (20.56) *0.37*	72.80 (22.29) *0.56*	63.30 (22.90) *1.05*	52.09 (21.97) *1.63*	a > g, f, j, i, h, k, d***; b, c, g, f, i, h > j***; c > i, k***; c > g**; c > f*; b > g, i, k***; b > f, h**; g, f, i, h, d > k***; j > k***; e > j, i, k***; e > g*; d > j**; b > d**
School Functioning	78.83 (19.59)	73.09 (20.35) *0.29*	70.62 (19.37) *0.42*	76.60 (17.00) 0.11	67.44 (20.06) *0.58*	63.18 (21.39) *0.80*	62.36 (20.61) *0.84*	70.61 (22.43) *0.42*	66.16 (23.33) *0.65*	62.40 (18.20) *0.84*	51.98 (21.41) *1.37*	a > b, c, g, f, j, i, h, k, e***; b, c, h > j***; b, c, g, f, j, i, h, d, e > k***; c > g**; c > f*; b > g, i***; b > f**; h > g***; h > f, i*; b, d > e*; d > g, j***; d > f**

Note: *p < .05, **p < .01, ***p < .001 based on analysis of variance with Tukey post-hoc tests.

With a Bonferroni correction for the number of comparisons, p < .005 values should be considered statistically significant.

Higher values equal better health-related quality of life. Disease clusters are ordered from left to right by decreasing overall HRQOL.

ES equals effect size.

Effect size represents the magnitude in the difference between the disease cluster and the healthy children sample.

Effect sizes are designated as small (.20), medium (.50), and large (.80).

Diabetes. Card. equals Cardiac. ESRD equals End Stage Renal Disease. Psych. equals Psychiatric Disorders s Rheumatology. CP equals Cerebral Palsy. GI equals Gastrointestinal Conditions.

#### Physical health

Patients across the disease clusters, with the exception of the diabetes cluster, self-reported significantly lower physical health in comparison to healthy children (Table 2). Parents across the disease clusters, with the exception of the diabetes and cardiac clusters, proxy-reported significantly lower patient physical health in comparison to healthy children (Table 3). The majority of differences for patient-report and parent proxy-report demonstrated medium to large effect sizes. Patients in the diabetes cluster self-reported the highest physical health, while parents proxy-reported the highest physical health for the cardiac cluster. Patients and parents in the cerebral palsy cluster reported the lowest overall physical health.

#### Psychosocial health

Patients and parents across the disease clusters reported significantly lower overall psychosocial health in comparison to healthy children and their parents (Tables 2 and 3). The majority of differences for patient-report and parent proxy-report demonstrate medium to large effect sizes. Patients in the diabetes cluster self-reported the highest overall psychosocial health, while parents proxy-reported the highest overall psychosocial health for the cardiac cluster. Patients in the psychiatric cluster self-reported the lowest psychosocial health, while parents proxy-reported the lowest overall psychosocial health for the cerebral palsy and psychiatric clusters.

#### Emotional functioning

Patients across the disease clusters, with the exception of the end stage renal disease cluster, self-reported significantly lower emotional functioning in comparison to healthy children (Table 2). The majority of differences demonstrate small to medium effect sizes. Parents across the disease clusters proxy-reported significantly lower patient emotional functioning in comparison to healthy children (Table 3). The majority of differences demonstrate large effect sizes. Patients in the end stage renal disease cluster self-reported the highest emotional functioning, while parents proxy-reported the highest emotional functioning for the cardiac cluster. Patients and parents in the psychiatric cluster reported the lowest emotional functioning.

#### Social functioning

Patients across the disease clusters, with the exception of the diabetes and gastrointestinal clusters, self-reported significantly lower social functioning in comparison to healthy children (Table 2). The majority of differences demonstrate small to medium effect sizes. Parents across the disease clusters, with the exception of the diabetes, cardiac, and gastrointestinal clusters, proxy-reported significantly lower patient social functioning in comparison to healthy children (Table 3). The majority of these differences demonstrate medium to large effect sizes. Patients in the diabetes cluster self-reported the highest social functioning, while parents proxy-reported the highest social functioning for the cardiac cluster. Patients and parents in the cerebral palsy cluster reported the lowest social functioning.

#### School Functioning

Patients and parents across the disease clusters, with the exception of the obesity cluster, reported significantly lower school functioning in comparison to healthy children and their parents (Tables 2 and 3). The majority of differences demonstrate medium to large effect sizes. Patients in the obesity and diabetes clusters self-reported the highest school functioning, while parents proxy-reported the highest school functioning for the obesity cluster. Patients and parents in the cerebral palsy cluster reported the lowest overall school functioning.

### Comparison of disease categories within disease clusters and disease severity within disease categories

#### Pediatric asthma

Patients with moderate to severe persistent asthma self-reported significantly lower overall HRQOL, physical health, psychosocial health, emotional functioning, and school functioning in comparison to patients with mild intermittent and mild persistent asthma (Table 4). Patients with moderate to severe persistent asthma self-reported significantly lower social functioning in comparison to patients with mild intermittent asthma. Patients with mild intermittent asthma self-reported the highest overall HRQOL.

**Table 4:** Child Self-Report: Means and Standard Deviations for the PedsQL™ 4.0 Generic Core Scales by Disease Category.

		Total Score	Physical Health	Psychosocial Health	Emotional Functioning	Social Functioning	School Functioning
	n	Mean (SD)	Mean (SD)	Mean (SD)	Mean (SD)	Mean (SD)	Mean (SD)

**Asthma**							
Mild Intermittent^a^	75	81.25 (12.68)	85.80 (16.81)	78.99 (14.58)	75.08 (19.24)	83.44 (21.60)	79.67 (20.15)
Mild Persistent^b^	28	73.71 (12.51)	77.95 (16.40)	71.61 (14.64)	70.09 (17.04)	78.12 (19.20)	67.26 (22.90)
Moderate to Severe Persistent ^c^	23	59.64 (21.56)	59.57 (23.50)	59.76 (23.09)	49.18 (27.59)	70.65 (27.28)	61.93 (32.74)
Differences		a > c***; b > c**	a > c***; b > c**	a > c***; b > c*	a > c***; b > c**	a > c*	a > b*; a > c**
**Cancer**							
Acute Lymphoblastic Leukemia^a^	109	73.25 (16.78)	74.16 (20.59)	72.86 (17.12)	72.57 (22.98)	76.90 (20.11)	68.67 (19.88)
Brain Tumor ^b^	194	71.04 (16.05)	71.29 (21.05)	71.02 (16.50)	72.32 (20.17)	73.00 (22.57)	67.84 (19.67)
Newly Diagnosed On-Treatment^¤^	105	68.92 (15.97)	65.54 (23.14)	71.04 (15.17)	68.81 (21.24)	77.19 (18.29)	66.22 (19.60)
Long-Term Survivor Off-Treatment > 12 months^§^	72	77.50 (15.30)	79.95 (18.78)	76.30 (16.04)	77.57 (20.31)	79.36 (19.77)	71.11 (18.11)
Differences		§ > ¤***	§ > ¤***	§ > ¤*	§ > ¤**	_____	_____
**Cardiac**							
Disease Severity Group 1 ^a^	52	82.67 (13.98)	87.97 (13.46)	79.82 (16.28)	76.15 (21.95)	83.46 (19.24)	79.31 (16.28)
Disease Severity Group 2 ^b^	92	76.30 (13.89)	81.45 (15.30)	73.56 (14.73)	72.09 (17.40)	77.80 (18.22)	70.82 (19.45)
Disease Severity Group 3 ^c^	63	76.36 (15.48)	80.82 (15.66)	73.92 (18.21)	72.46 (20.79)	77.92 (21.80)	71.43 (21.30)
Disease Severity Group 4 ^d^	78	75.81 (13.93)	78.42 (17.69)	74.41 (15.01)	74.68 (21.02)	77.66 (19.03)	70.96 (18.15)
Differences		a > d*	a > d**	_____	_____	_____	_____
**Cerebral Palsy**							
Hemiplegia^a^	30	73.14 (12.81)	75.41 (16.97)	71.78 (13.83)	72.80 (23.43)	73.00 (17.40)	70.56 (17.02)
Diplegia^b^	29	68.71 (16.55)	63.95 (19.66)	71.17 (17.12)	72.50 (20.75)	73.10 (20.76)	67.93 (24.04)
Paraplegia^c^	11	53.86 (12.21)	45.70 (16.55)	58.20 (14.42)	57.73 (16.64)	60.00 (17.89)	57.27 (21.84)
Differences		a > c**; b > c*	a > b*; a > c***;b > c*	a > c*	_____	_____	_____
**Diabetes**							
Type 1 ^a^	209	81.64 (12.44)	86.66 (13.21)	78.90 (14.09)	73.99 (19.49)	86.42 (15.81)	76.59 (16.68)
Type 2 ^b^	88	77.46 (13.68)	84.36 (13.62)	73.75 (15.52)	68.69 (19.54)	83.47 (17.29)	68.97 (20.23)
Differences		a > b*	_____	a > b**	a > b*	_____	a > b**
**End Stage Renal Disease**							
Transplant^a^	39	78.94 (14.24)	80.76 (20.53)	77.86 (13.46)	79.04 (18.70)	82.31 (17.54)	72.31 (16.13)
Peritoneal Dialysis^b^	15	70.70 (16.53)	72.89 (15.33)	69.69 (18.66)	68.75 (22.74)	73.50 (24.38)	66.25 (18.28)
Hemodialysis^c^	31	69.30 (14.29)	68.04 (20.74)	69.95 (13.29)	73.39 (16.45)	76.01 (13.46)	60.48 (21.46)
Differences		a > c*	a > c*	_____	_____	_____	_____
**Gastrointestinal Conditions**							
Irritable Bowel Syndrome^a^	119	77.90 (12.64)	80.49 (13.23)	76.50 (13.81)	74.93 (17.43)	84.09 (15.43)	70.59 (18.70)
Functional Abdominal Pain^b^	81	79.98 (10.62)	82.83 (11.29)	78.46 (12.10)	75.49 (17.06)	86.17 (13.28)	73.70 (15.96)
Organic Disorders^c^	80	75.41 (16.01)	79.21 (16.70)	73.39 (17.60)	70.94 (21.93)	82.81 (18.40)	66.39 (20.23)
Differences		_____	_____	_____	_____	_____	b > c*
**Obesity**							
Severely Obese^a^	106	67.00 (16.30)	71.00 (18.80)	64.90 (17.70)	63.20 (20.10)	67.50 (25.00)	64.10 (20.40)
Obese^b^	63	74.00 (14.20)	77.50 (17.90)	72.10 (14.10)	68.60 (18.50)	72.60 (18.20)	75.00 (14.50)
Overweight^c^	294	79.30 (12.80)	83.50 (13.00)	77.00 (14.00)	72.60 (17.70)	80.20 (16.60)	78.30 (15.50)
Differences		b > a**; c > a***; c > b**	c > b**; c > a***; b > a*	b > a**; c > a***; c > b*	c > a***	c > a***; c > b**	c > a***; b > a**
**Psychiatric Disorders**							
Attention-Deficit and Disruptive Behavior Disorders^a^	107	72.40 (12.20)	84.30 (13.20)	66.00 (14.70)	61.30 (19.50)	70.30 (21.40)	66.40 (16.20)
Anxiety Disorders^b^	57	71.30 (12.20)	78.60 (15.60)	67.40 (13.60)	59.00 (17.70)	74.40 (19.50)	68.90 (13.90)
Mood Disorders ^c^	29	69.70 (13.10)	80.00 (13.30)	64.20 (15.00)	55.50 (21.70)	74.40 (22.00)	63.90 (16.40)
Pervasive Developmental Disorders ^d^	28	69.60 (14.00)	78.50 (14.30)	64.80 (16.60)	63.80 (17.40)	63.10 (23.00)	67.60 (20.50)
Differences		_____	_____	_____	_____	_____	_____
**Rheumatology**							
Systemic Lupus Erythematosus^a^	29	76.95 (14.97)	76.13 (19.81)	77.46 (15.69)	70.65 (23.70)	89.44 (14.29)	73.52 (16.63)
Spondyloarthritis^b^	39	74.92 (18.41)	69.24 (25.10)	77.99 (17.07)	77.27 (22.24)	84.87 (19.54)	72.18 (20.96)
Juvenile Rheumatoid Arthritis^c^	100	73.73 (16.47)	70.88 (21.52)	75.18 (16.65)	73.13 (20.87)	78.64 (19.61)	74.27 (19.07)
Fibromyalgia^d^	57	55.86 (15.16)	45.40 (19.86)	61.44 (16.25)	53.77 (22.41)	74.47 (18.21)	55.74 (20.46)
Differences		a, b, c > d***	a, b, c > d***	a, b, c > d***	b, c > d***; a > d*	a > d**	c > d***; a, b > d**

Note: *p < .05, **p < .01, ***p < .001 based on Tukey post-hoc analysis when more than 2 disease categories are being compared and independent samples t-test when 2 disease categories are being compared. Contrasts for cancer disease categories are for Acute Lymphoblastic Leukemia^a ^versus Brain Tumor^b^and Newly Diagnosed On-Treatment ^¤ ^versus Long Term Survivor Off-Treatment > 12 months ^§^. Asthma contrasts are based on the PedsQL™ 4.0 SF15, a shorted version of the 23-item PedsQL™ 4.0 Generic Core Scales.

Parents of patients with mild persistent and moderate to severe persistent asthma proxy-reported significantly lower patient overall HRQOL, physical health, psychosocial health, emotional functioning, and school functioning in comparison to patients with mild intermittent asthma (Table 5). Parents of patients with moderate to severe persistent asthma proxy-reported significantly lower social functioning in comparison to patients with mild intermittent asthma. Parents of patients with mild intermittent asthma proxy-reported the highest overall patient HRQOL.

**Table 5:** Parent Proxy-Report: Means and Standard Deviations for the PedsQL™ 4.0 Generic Core Scales by Disease Category.

		Total Score	Physical Health	Psychosocial Health	Emotional Functioning	Social Functioning	School Functioning
	n	Mean (SD)	Mean (SD)	Mean (SD)	Mean (SD)	Mean (SD)	Mean (SD)

**Asthma**							
Mild Intermittent^a^	206	86.90 (13.23)	88.80 (14.37)	85.86 (15.63)	85.60 (17.84)	88.43 (17.26)	83.02 (20.97)
Mild Persistent^b^	68	76.77 (16.23)	76.15 (21.40)	77.11 (16.74)	74.54 (20.58)	82.23 (18.71)	75.00 (22.76)
Moderate to Severe Persistent ^c^	63	69.88 (16.82)	64.76 (21.93)	72.65 (18.10)	69.84 (22.30)	76.85 (24.80)	71.39 (23.34)
Differences		a > b, c***; b > c*	a > b, c***; b > c**	a > b, c***	a > b, c***	a > c***	a > b*; a > c**
**Cancer**							
Acute Lymphoblastic Leukemia^a^	170	71.29 (18.11)	71.20 (22.54)	71.35 (17.90)	67.57 (19.97)	77.00 (20.40)	68.03 (22.51)
Brain Tumor ^b^	249	66.21 (19.22)	65.17 (25.24)	67.01 (18.89)	67.34 (21.11)	68.43 (23.92)	65.12 (23.53)
Newly Diagnosed On-Treatment^¤^	180	66.95 (19.85)	65.00 (26.26)	68.19 (18.25)	63.26 (20.70)	75.58 (20.08)	63.61 (24.03)
Long-Term Survivor Off-Treatment > 12 months^§^	94	73.64 (18.72)	74.80 (24.08)	72.95 (18.07)	72.93 (19.94)	76.28 (21.71)	68.87 (23.08)
Differences		a > b**;§ > ¤**	a > b*; § > ¤**	a > b*; § > ¤*	§ > ¤***	a > b***	_____
**Cardiac**							
Disease Severity Group 1 ^a^	82	88.06 (12.04)	93.03 (10.39)	84.98 (14.63)	81.05 (19.23)	91.38 (14.19)	80.86 (17.88)
Disease Severity Group 2 ^b^	143	80.50 (15.13)	84.32 (18.39)	78.28 (15.58)	75.78 (20.12)	84.55 (18.73)	72.68 (19.54)
Disease Severity Group 3 ^c^	93	78.62 (16.21)	81.86 (18.63)	76.92 (17.57)	74.50 (20.97)	81.66 (20.40)	72.46 (20.36)
Disease Severity Group 4 ^d^	108	73.42 (18.11)	75.37 (21.35)	72.41 (18.49)	70.10 (21.29)	76.17 (22.09)	68.68 (23.22)
Differences		a > b**; a > c, d***; b > d**	b > d**; a > b**; a > c, d***	a > b*; a > c**; a > d***; b > d*	a > d**	a > c**; b > d**; a > d***	a > b*; a > d**
**Cerebral Palsy**							
Hemiplegia^a^	49	57.67 (20.00)	55.72 (25.78)	58.94 (19.45)	62.19 (21.71)	55.92 (24.02)	57.26 (22.56)
Diplegia^b^	67	54.03 (15.87)	47.02 (22.15)	58.01 (15.97)	63.31 (19.32)	53.88 (20.29)	56.39 (20.40)
Paraplegia^c^	73	44.78 (15.30)	31.88 (27.19)	52.00 (14.80)	61.31 (17.61)	48.29 (20.62)	45.24 (19.65)
Differences		a > c***; b > c**	a > c***; b > c**	_____	_____	_____	a, b > c**
**Diabetes**							
Type 1 ^a^	226	77.31 (14.12)	82.38 (17.82)	74.53 (15.17)	69.11 (18.64)	82.33 (18.78)	71.87 (19.11)
Type 2 ^b^	78	74.71 (14.01)	81.06 (15.64)	71.28 (15.77)	69.47 (18.26)	77.21 (20.70)	67.42 (20.14)
Differences		_____	_____	_____	_____	a > b*	_____
**End Stage Renal Disease**							
Transplant^a^	45	75.57 (17.75)	78.38 (24.65)	74.14 (17.50)	75.67 (18.36)	78.56 (22.20)	66.06 (18.53)
Peritoneal Dialysis^b^	19	68.07 (15.02)	68.40 (20.01)	67.97 (14.79)	62.11 (17.90)	73.42 (17.95)	69.30 (16.21)
Hemodialysis^c^	31	61.94 (17.70)	62.52 (23.45)	61.69 (17.23)	62.74 (19.79)	66.13 (19.27)	56.21 (25.62)
Differences		a > c**	a > c*	a > c**	a > b, c*	a > c*	_____
**Gastrointestinal Conditions**							
Irritable Bowel Syndrome^a^	122	74.82 (14.27)	78.68 (15.83)	72.72 (15.30)	66.19 (19.61)	82.54 (17.34)	69.35 (20.48)
Functional Abdominal Pain^b^	82	73.65 (12.23)	77.70 (15.41)	71.49 (12.87)	65.49 (16.59)	79.94 (15.20)	68.93 (17.99)
Organic Disorders^c^	82	68.75 (16.97)	70.31 (18.82)	67.95 (18.03)	63.41 (21.30)	77.33 (21.29)	63.13 (20.96)
Differences		a > c*	a > c*	_____	_____	_____	_____
**Obesity**							
Severely Obese^a^	105	63.30 (19.20)	63.60 (24.00)	63.10 (18.60)	60.90 (21.70)	67.20 (26.10)	61.40 (21.50)
Obese^b^	63	75.00 (14.50)	76.30 (17.60)	73.90 (15.30)	72.60 (17.80)	73.50 (17.30)	76.60 (17.00)
Overweight^c^	294	80.00 (13.60)	82.60 (17.20)	76.10 (14.40)	74.90 (15.80)	82.30 (16.10)	78.40 (17.10)
Differences		b, c > a***; c > b**	c > b**; c > a***; b > a**	b, c > a***	c > a***; b > a**	c > a***; c > b**	b, c > a***
**Psychiatric Disorders**							
Attention-Deficit and Disruptive Behavior Disorders^a^	107	65.80 (13.50)	80.30 (16.20)	58.00 (15.40)	54.90 (18.70)	59.10 (22.50)	59.80 (17.80)
Anxiety Disorders^b^	57	66.00 (14.20)	80.30 (17.60)	58.20 (15.10)	46.80 (16.70)	66.30 (25.70)	62.00 (17.80)
Mood Disorders ^c^	29	65.70 (11.70)	79.20 (17.80)	58.60 (12.30)	49.10 (16.90)	66.20 (18.40)	60.60 (16.70)
Pervasive Developmental Disorders ^d^	28	61.50 (13.10)	76.30 (22.00)	53.70 (12.40)	53.70 (14.00)	47.20 (20.20)	61.40 (15.80)
Differences		_____	_____	_____	a > b**	_____	_____
**Rheumatology**							
Systemic Lupus Erythematosus^a^	18	75.25 (17.69)	70.16 (25.35)	78.00 (15.67)	72.50 (21.71)	83.61 (16.79)	78.53 (17.30)
Spondyloarthritis^b^	33	73.63 (18.20)	70.08 (27.65)	75.49 (15.31)	75.00 (17.94)	81.09 (19.17)	70.61 (18.91)
Juvenile Rheumatoid Arthritis^c^	124	73.48 (18.95)	70.07 (24.07)	75.36 (18.13)	74.58 (19.89)	76.73 (20.89)	74.66 (22.50)
Fibromyalgia^d^	57	52.07 (15.17)	43.37 (19.01)	56.70 (16.41)	48.95 (19.47)	67.02 (19.70)	53.77 (20.55)
Differences		a, b, c > d***	a, b, c > d***	a, b, c > d***	a, b, c > d***	a, b, c > d*	a, b > d**; c > d***

Note: *p < .05, **p < .01, ***p < .001 based on Tukey post-hoc analysis when more than 2 disease categories are being compared and independent samples t-test when 2 disease categories are being compared. Contrasts for cancer disease categories are for Acute Lymphoblastic Leukemia^a ^versus Brain Tumor^b^and Newly Diagnosed On-Treatment ^¤ ^versus Long Term Survivor Off-Treatment > 12 months ^§^. Asthma contrasts are based on the PedsQL™ 4.0 SF15, a shorted version of the 23-item PedsQL™ 4.0 Generic Core Scales.

#### Pediatric cancer

Disease category comparisons in the cancer cluster were between the two most prevalent forms of pediatric cancer, acute lymphoblastic leukemia and brain tumors. Patients with acute lymphoblastic leukemia self-reported no significant HRQOL differences compared to patients with brain tumors (Table 4). Patients with newly-diagnosed cancer on-treatment versus long-term survivors off-treatment > 12 months were also compared. Patients newly-diagnosed receiving cancer treatment self-reported significantly lower overall HRQOL, physical health, psychosocial health, and emotional functioning in comparison to long-term survivors.

Parents of patients with brain tumors proxy-reported significantly lower overall HRQOL, physical health, psychosocial health, and social functioning in comparison to pediatric patients with acute lymphoblastic leukemia (Table 5). Parents of patients with newly-diagnosed cancer on-treatment reported significantly lower overall HRQOL, physical health, psychosocial health, and emotional functioning in comparison to long-term survivors.

#### Pediatric cardiac conditions

Patients in disease severity group 4 self-reported significantly lower overall HRQOL in comparison to patients in disease severity group 1. Patients in disease severity group 4 self-reported significantly lower physical health in comparison to patients in disease severity group 1 (Table 4).

Parents of patients in disease severity group 4 proxy-reported significantly lower overall HRQOL, physical health, psychosocial health, emotional functioning, social functioning, and school functioning in comparison to patients in disease severity group 1 (Table 5). Parents of patients in disease severity group 4 proxy-reported significantly lower overall HRQOL, physical health, psychosocial health, and social functioning in comparison to patients in disease severity group 2. Parents of patients in disease severity group 2 proxy-reported significantly lower overall HRQOL, physical health, psychosocial health, and school functioning in comparison to patients in disease severity group 1. Parents of patients in disease severity group 3 proxy-reported significantly lower overall HRQOL, physical health, psychosocial health, and social functioning in comparison to patients in disease severity group 1.

#### Pediatric cerebral palsy

Patients with paraplegia self-reported significantly lower overall HRQOL, physical health, and psychosocial health in comparison to patients with hemiplegia (Table 4). Patients with paraplegia self-reported significantly lower overall HRQOL and physical health in comparison to patients with diplegia. Patients with diplegia self-reported significantly lower physical health in comparison to patients with hemiplegia.

Parents of patients with paraplegia proxy-reported significantly lower overall HRQOL, physical health, and school functioning in comparison to pediatric patients with hemiplegia and diplegia (Table 5).

#### Pediatric diabetes

Patients with Type 2 diabetes self-reported significantly lower overall HRQOL, psychosocial health, emotional functioning, and school functioning in comparison to patients with Type 1 diabetes (Table 4).

Parents of patients with Type 2 diabetes proxy-reported significantly lower social functioning in comparison to patients with Type 1 diabetes (Table 5).

#### Pediatric end stage renal disease

Patients on hemodialysis self-reported significantly lower overall HRQOL and physical health in comparison to patients with renal transplants (Table 4).

Parents of patients on hemodialysis proxy-reported significantly lower overall HRQOL, physical health, psychosocial health, emotional functioning, and social functioning in comparison to patients with renal transplants (Table 5). Parents of patients on peritoneal dialysis proxy-reported significantly lower emotional functioning in comparison to patients with renal transplants.

#### Pediatric gastrointestinal conditions

Patients with organic gastrointestinal disorders self-reported significantly lower school functioning in comparison to patients with functional abdominal pain (Table 4).

Parents of patients with organic gastrointestinal disorders reported significantly lower overall HRQOL and physical health in comparison to patients with irritable bowel syndrome (Table 5).

#### Pediatric obesity

Severely obese children self-reported significantly lower overall HRQOL, physical health, psychosocial health, and school functioning in comparison to obese children (Table 4). Severely obese children self-reported significantly lower overall HRQOL, physical health, psychosocial health, emotional functioning, social functioning, and school functioning in comparison to overweight children. Obese children self-reported significantly lower overall HRQOL, physical health, psychosocial health, and social functioning in comparison to overweight children.

Parents of severely obese children proxy-reported significantly lower overall HRQOL, physical health, psychosocial health, emotional functioning, and school functioning in comparison to obese children (Table 5). Parents of severely obese children proxy-reported significantly lower overall HRQOL, physical health, psychosocial health, emotional functioning, social functioning, and school functioning in comparison to overweight children. Parents of obese children proxy-reported significantly lower overall HRQOL, physical health, and social functioning in comparison to overweight children.

#### Pediatric psychiatric disorders

Patients with psychiatric disorders self-reported no statistically significant differences (Table 4).

Parents of patients with anxiety disorders proxy-reported significantly lower emotional functioning in comparison to patients with attention-deficit/disruptive behavior disorders (Table 5).

#### Pediatric rheumatology

Patients with fibromyalgia self-reported lower overall HRQOL, physical health, psychosocial health, emotional functioning, and school functioning in comparison to other rheumatologic conditions (Table 4). Patients with fibromyalgia self-reported lower social functioning in comparison to patients with systemic lupus erythematosus.

Parents of patients with fibromyalgia proxy-reported lower overall HRQOL, physical health, psychosocial health, emotional functioning, social functioning, and school functioning in comparison to other rheumatologic conditions (Table 5).

## Discussion

The results demonstrate differential effects of pediatric chronic conditions on patient HRQOL across ten pediatric chronic disease clusters and 33 disease categories/severities from the perspectives of pediatric patients and parents utilizing the PedsQL™ 4.0 Generic Core Scales. The present study on over 2,500 pediatric patients from 10 physician-diagnosed disease clusters and 33 disease categories/severities represents the largest single investigation of generic HRQOL across pediatric chronic health conditions that we are aware of, and as such, makes an important contribution to the extant literature on pediatric chronic health conditions.

In the present study, pediatric patients with diabetes, gastrointestinal conditions, cardiac conditions, asthma, obesity, end stage renal disease, psychiatric disorders, cancer, rheumatologic conditions, and cerebral palsy self-reported progressively more impaired overall HRQOL than healthy children, respectively, with medium to large effect sizes. Patients with cerebral palsy self-reported the most impaired HRQOL, while patients with diabetes self-reported the best HRQOL. Parent proxy-reports generally paralleled patient self-report, with several notable differences. Parents proxy-reported cardiac conditions, diabetes, obesity, gastrointestinal conditions, end stage renal disease, asthma, rheumatologic conditions, cancer, psychiatric conditions, and cerebral palsy as having progressively more impaired overall HRQOL than healthy children, respectively, with medium to large effect sizes. Given the well documented evidence in both the adult and pediatric literature that information provided by proxy-respondents is not equivalent to that reported by the patient [40,41], the imperfect agreement between pediatric patient and parent perspectives on the magnitude of impairment across the pediatric chronic conditions in the current investigation is not an unexpected finding.

The comparisons between pediatric patients with chronic health conditions with healthy children's PedsQL™ scores are useful in understanding the relative clinical impact of different pediatric chronic health conditions on HRQOL. The extant literature on the adaptation of children with chronic physical health conditions demonstrates that children with chronic physical health conditions are reported to not only experience lower physical functioning, but also manifest lower emotional, social, and school functioning in comparison to healthy children [42]. The findings from the present study indicates substantial differences on the impact of specific chronic conditions on HRQOL, with differential impacts on physical, emotional, social, and school functioning.

The challenge for health care is to identify and enroll pediatric patients with chronic health conditions in high quality evidence-based comprehensive healthcare services in order to mitigate the potential long-term negative consequences on patient HRQOL. For some chronic health conditions such as fibromyalgia, HRQOL and symptom scales completed by patients may be the only indicators of disease activity and treatment effect [43-45]. In such patient populations, patient-reported HRQOL is often indicated as the primary end-points for clinical trials [43-45]. In clinical practice, patient-reported HRQOL should be a component of high quality comprehensive care [3].

There are a number of potential limitations of the study. Working from an existing database, several disease categories and severities contained small sample sizes, attenuating the statistical power to find significant differences. Given the rather low prevalence rate of most pediatric chronic conditions, we did not have the sample size to match samples for age, gender, race/ethnicity, and SES. Given the requirements of the local IRBs, information on nonparticipants was not available, nor was the nonresponse rates across samples, which may limit the generalizability of the findings. Finally, we did not match the pediatric patient and parent proxy-report databases as parent/child dyads since we anticipated that there would circumstances when children were too young (i.e., under age 5), too cognitively impaired, too ill or fatigued to complete a HRQOL instrument, and consequently, there would be individual patients and groups of patients (e.g., pediatric patients with cerebral palsy) in which parent proxy-report at the group level would be lower for some dimensions given that the more severely impaired or ill children would not being able to self-report. Future research is indicated to investigate the various chronic conditions under which pediatric patient self-report and parent proxy-report are most discrepant, and the reasons why this discrepancy might occur.

## Conclusion

The data contained within this study represents a larger and more diverse population of pediatric patients with chronic conditions than previously reported in the extant literature. The findings contribute important information on the differential effects of pediatric chronic conditions on generic HRQOL from the perspectives of children and parents utilizing the PedsQL™ 4.0 Generic Core Scales. These findings with the PedsQL™ have clinical implications for the healthcare services provided for children with chronic health conditions. Given the degree of reported impairment based on PedsQL™ scores across different pediatric chronic conditions, the need for more efficacious targeted treatments for those pediatric patients with more severely impaired HRQOL is clearly and urgently indicated.

## Abbreviations

HRQOL Health-Related Quality of Life

PedsQL™ Pediatric Quality of Life Inventory™

## Competing interests

Dr. Varni holds the copyright and the trademark for the PedsQL™ and receives financial compensation from the Mapi Research Trust, which is a nonprofit research institute that charges distribution fees to for-profit companies that use the Pediatric Quality of Life Inventory™. The PedsQL™ is available at the PedsQL™ Website [46].

## Authors' contributions

JWV conceptualized the rationale and design of the study. JWV and CAL drafted the manuscript. CAL performed the statistical analyses. TMB participated in the statistical analyses. All authors read and approved the final manuscript.
